# Comparative Effectiveness of Enhanced Recovery After Surgery Program Combined With Single-Incision Laparoscopic Surgery in Colorectal Cancer Surgery: A Retrospective Analysis

**DOI:** 10.3389/fonc.2021.768299

**Published:** 2022-01-12

**Authors:** Changgang Wang, Haoran Feng, Xiaoning Zhu, Zijia Song, You Li, Yiqing Shi, Yimei Jiang, Xianze Chen, Tao Zhang, Ren Zhao, Kun Liu

**Affiliations:** ^1^ Department of General Surgery, Ruijin Hospital, Shanghai Jiaotong University School of Medicine, Shanghai, China; ^2^ Department of Neurology Department, Ruijin Hospital, Shanghai Jiaotong University School of Medicine, Shanghai, China

**Keywords:** ERAS, SILS, colorectal cancer, retrospective analysis, CLS

## Abstract

**Background:**

Recently, enhanced recovery after surgery (ERAS) has been widely used in the perioperative management of colorectal cancer (CRC). This study aimed to evaluate the safety and feasibility of ERAS combined with single-incision laparoscopic surgery (SILS) in CRC surgery.

**Methods:**

This was a retrospective study of patients with CRC who underwent surgery between April 2018 and April 2020 in Ruijin Hospital(North), Shanghai Jiaotong University School of Medicine. The patients were divided into three groups: group A (n=138), patients who underwent traditional multiport laparoscopic colectomy with conventional perioperative management; group B (n=63), patients who underwent SILS; and group C (n=51), patients who underwent SILS with ERAS.

**Results:**

Overall, 252 participants were included in the retrospective study. The median operation time (min) in group B and group C was shorter than that in group A (group A 134.0 ± 42.5; group B 117 ± 38.9; group C 111.7 ± 35.4, p=0.004). The estimated surgical blood loss (ml) was lower in groups B and C than in group A (group A 165.1 ± 142.2; group B 122.0 ± 79.4; group C 105.2 ± 55.8, p=0.011). The length of surgical incision (cm) was shorter in groups B and C than in group A (group A 7.34 ± 1.05; group B 5.60 ± 0.80; group C 5.28 ± 0.52, p<0.001). The time before first flatus (hours) in group C was shorter than in groups A and B (group A 61.85 ± 21.14; group B 58.30 ± 20.08; group C 42.06 ± 23.72; p<0.001). The days prior to the administration of free oral fluids in group C was shorter than in groups A and B (group A 4.79 ± 1.28; group B 4.67 ± 1.11; group C 2.62 ± 0.64; p<0.001). The days of prior solid diet was less in group C than in groups A and B (group A 7.22 ± 3.87; group B 7.08 ± 3.18; group C 5.75 ± 1.70; p=0.027). The postoperative length of stay (LOS) was less in group C compared with that in groups A and B (group A 9.46 ± 4.84 days; group B 9.52 ± 7.45 days; group C 7.20 ± 2.37 days; p=0.023). The visual analog scale (VAS) scores on day 0, 1, and 2 in groups B and C were lower than those in group A (day 0, p<0.001; day 1, p<0.001; day 2, p=0.002), while the VAS score on day 3 showed no differences in the three groups (group A 1.29 ± 1.38; group B 0.98 ± 1.24; group C 0.75 ± 0.64, p=0.018).

**Conclusion:**

The findings suggest that SILS combined with ERAS may be a feasible and safe procedure for CRC surgery because it provides favorable cosmetic results, early dietary resumption, shorter hospital stays, and appropriate control of postoperative pain without increases in complications or readmission rates compared to conventional perioperative care with SILS or conventional laparoscopic surgery(CLS) of CRC. Further prospective randomized controlled studies are needed to enhance evidence-based medical evidence.

## Introduction

According to global cancer statistics, colorectal cancer (CRC) is the third most common cancer worldwide and the second leading cause of mortality (1).With the rapid development of laparoscopic technology and instruments, laparoscopic radical resection of CRC has been proven safe and effective in multiple randomized controlled trials compared with traditional open surgery (2–5). Moreover, laparoscopic surgery has many advantages, such as less trauma, good cosmetic effect, less postoperative pain, and shorter hospital stay (6–8).Single incision laparoscopic colorectal surgery (SILS) has been developed based on traditional laparoscopic surgery. It was first reported in 2008 that Bucher et al. (9) successfully performed a single-hole laparoscopic right hemicolectomy for a patient with colonic polyps and achieved good results. Since our colorectal center performed the first single-incision laparoscopic radical resection of CRC in 2013, more than 400 single-incision laparoscopic radical resections of CRC have been performed, including right hemicolectomy, left hemicolectomy, sigmoid hemicolectomy, and high rectal surgery. Through retrospective analysis and prospective RCT research, it was confirmed that the safety and curative effects of single-incision laparoscopic radical resection of CRC are not inferior to those of traditional laparoscopic surgery (10, 11).

Since its introduction in 1997 by Professor Kehlet (12), enhanced recovery after surgery (ERAS) has achieved great success in clinical practice worldwide and has shown advantages in CRC surgery. Using such a multimodal stress-minimizing approach has been shown to reduce rates of morbidity, improve recovery, and shorten the length of stay (LOS) after a major colorectal surgery (13).

However, the effectiveness of the ERAS program combined with SILS in CRC surgery is unclear. Few clinical studies have reported the effectiveness of the ERAS program combined with SILS in CRC surgery (14). In this study, we performed a retrospective analysis to evaluate the effect of the ERAS program with SILS in CRC surgery.

## Methods

### Patients

A retrospective cohort study was performed on 252 patients who underwent SILS or traditional laparoscopic surgery for CRC at the Department of Colorectal Surgery, Ruijn Hospital(North), Shanghai Jiaotong University School of Medicine from April 2018 to April 2020. All patients were divided into three groups: group A (n=138), patients who underwent traditional multiport laparoscopic colorectal surgery with conventional perioperative management; group B (n=63), patients who underwent SILS; and group C (n=51), patients who underwent SILS with the ERAS concept. The study was approved by the local research ethics committee of Ruijn Hospital(North), Shanghai Jiaotong University School of Medicine and followed the international and national regulations in accordance with the Declaration of Helsinki. A written informed consent was obtained from all patients, allowing us to store their data in our hospital database and use it for clinical research.

The inclusion criteria were as follows: (1) tumor clinical stage IA to IIIC according to the seventh edition of the American Joint Committee on Cancer (AJCC); (2) tumor diameter ≤ 5 cm; and (3) body mass index (BMI) ≤ 35 kg/m^2^. The exclusion criteria were as follows: (1) metastatic disease; (2) simultaneous or metachronous multiple cancers with disease-free survival ≤ 5 years; (3) simultaneous surgery for other diseases; (4) emergency operation; (5) neoadjuvant chemoradiotherapy; and (6) ASA IV or V according to the American Society of Anesthesiologists classification (ASA). The ERAS group had other exclusion criteria: (1) cT4b; and (2) tumor diameter ≥ 4 cm.

### Perioperative Management

Groups A and B were treated with traditional perioperative management, and group C was treated with ERAS according to the protocol of the ERAS Society. The perioperative protocols of the traditional method and ERAS are shown in **Table 1**.

**Table 1:** Difference of perioperative management between ERAS group and traditional control group.

Treatment Measures	ERAS Group	Traditional Control Group
Rehabilitation education	Anaesthesiologic, cardiologic and surgical counselling	No
Preoperative bowel preparation	Unconventional,Only for rectal resection	Preoperative enema the evening before surgery
Preoperative fasting	6 h, solid foods (inedible);2 h, Clear fluids,Carbohydrates oral loading(edible)	12 h
Nasogastric tub	Unconventional,Orogastric tube placed at the beginning of surgery, removed at the end of the procedure	Unlimited
Multi-modal anaesthetic protocol	General anesthesia, ultrasound-guided transversus abdominis plane block,Injection of local anaesthetics on the region of surgical wounds	General anesthesia
Prevention of intraoperative hypothermia	Warming device,Warmed intravenous fluids	Unconvention
Urinary drainage	1-2 d	3-5 d
Abdominal drainage	Often placed 1-2 d	3-5 d
Modes of postoperative analgesia	Injection of analgesic drugs other than opioids	Venous self-control analgesic pump( o pioid pain killers used)
Perioperative nutritional care	Nutritional screening highly recommended considering BMI and albumin level	Nutritional screening highly recommended considering BMI and albumin level
Early mobilisation	Full mobilization on the first postoperative day	Unlimited
Prophylaxis against thromboembolism	Compression stockings,LMWH according to Caprini score	Unlimited
Postoperative oral feeding	Light hospital diet and oral nutritional supplements on the first postoperative day, full hospital diet in the second postoperative day	Unlimited

### Surgical Procedures

Six qualified surgeons with over 50 cases of laparoscopic CRC surgery performed the operations in the conventional laparoscopic surgery (CLS) group. The SILS group operations were all performed by the same surgeon (Z.R.), who had performed over 200 cases of SILS for CRC.

After general anesthesia, the patients were placed in optimal positions according to the surgical approach. In general, straddle-type supine, Trendelenberg with left-tilted or right-tilted position was used in right colectomy or left colectomy, respectively. Additionally, modified lithotomy, Trendelenberg, right-tilted position was used in sigmoidectomy and anterior resection.

In the SILS group, a SILS™ Port (Covidien, Mansfield, MA, USA) with three 5-mm cannulas inserted or a Star-Port (Surgaid^®^, Guangzhou, China) consisting of three fixed instrument channels (one 5-mm, two 10-mm, and one 12-mm) was installed through a 2-3 cm midline periumbilical incision. A 30° laparoscope, a 0° flexible laparoscope (LTF-VP, Olympus Medical Systems, Tokyo, Japan), or an Olympus 3D laparoscope were used based on the choice of port. In cases using the SILS™ Port, the main operating cannula was changed from 5 mm to 12 mm when using Endo GIA™. In the CLS group, the operation was performed with 3-5 trocars, including a 12-mm trocar for a 30° laparoscope or a 3D laparoscope in the periumbilical area. The main operating trocar was 12-mm, while the remaining trocars was 5-mm. All surgeries in both groups were performed using conventional laparoscopic instruments.

All surgeries were performed according to the same oncologic principles, including complete mesocolic excision for colon cancer and total mesorectal excision for rectal cancer with D3 lymph node dissection. The medial-to-lateral or lateral-to-medial approach was adopted depending on the surgeon. For sigmoidectomy and anterior resection, mobilization of the splenic flexure was not performed routinely, except in cases of a lack of redundancy of the sigmoid colon or excessive anastomotic tension. Prophylactic ileostomy was performed depending on the anastomosis.

The specimen was retrieved through the wound protector installed through a transumbilical incision (SILS group) or a 3-4 cm additional incision (CLS group). The draining tube was extracted through the incision in the SILS group or through the main operating channel in the CLS group. The incisions were closed using an absorbable monofilament. Details of the surgical procedure were described in our previous reports (10, 11).

### Outcomes

The primary outcome was early morbidity, defined as postoperative complications observed within 30 days after surgery. It was graded according to the Clavien-Dindo classification. The secondary outcomes included intraoperative outcomes (operation time, estimated blood loss, incision length, and conversion rate), postoperative pain score, postoperative recovery (time to first ambulation, flatus, liquid diet and soft diet, LOS), and pathologic outcomes (tumor size, number of harvested lymph nodes, and proximal and distal resection margins). The incision length was defined as the sum of all the incision lengths. Postoperative pain was recorded using the visual analog scale (VAS) pain score (0-10 points) on postoperative day 0, 1, 2, and 3. Pathological outcomes were evaluated by pathologists. Follow-up was consistent with the National Comprehensive Cancer Network guidelines. Recurrence was confirmed using radiological and histological methods.

### Statistical Analyses

Statistical analyses were performed using SPSS (version 22.0, SPSS Inc. Chicago, IL, USA). Continuous variables were presented as mean ± standard deviation and categorical variables were described as numbers with percentages. The one-way analysis of variance (ANOVA) test were used for continuous variables of three groups, whereas proportions were compared using Pearson chi-square(χ2) test or Fisher’s exact test, as appropriate. All P values were 2-tailed, statistical significance was accepted for P values of <0.05.

## Results

### Baseline Characteristics and Types Of Surgeries

As shown in **Table 2**, there was no significant difference in terms of age, sex, BMI, preoperative serum CEA, and ASA grade among 3 groups. The types of surgeries are also shown in **Table 2**.

**Table 2:** Patient demographics, baseline characteristics, and type of operations performed according to tumor location.

Parameter	CLS	SILS	SILS (ERAS)	*P* value
Number of patients, n	138	63	51	
Age (years)				0.052
Mean ± SD	62.12 ± 12.09	60.84 ± 11.59	57.47±10.26	
Sex, n (%)				0.697
Males	88 (63.8)	44 (69.8)	33 (64.7)	
Females	50 (36.2)	19 (30.2)	18 (35.3)	
BMI (kg/m^2^)				0.596
Mean ± SD	23.40±3.07	23.40±3.07	23.40±3.07	
Preoperative serum CEA (ng/mL),n (%)				0.112
≤5	99 (71.7)	46 (73.0)	44 (86.3)	
>5	39 (28.3)	17 (27.0)	7 (13.7)	
ASA grade, n (%)				0.333
I	42 (30.4)	19 (30.2)	11 (21.6)	
II	80 (58.0)	35 (55.6)	37 (72.5)	
III	16 (11.6)	9 (14.3)	3 (5.9)	
Type of procedure, n (%)				–
Right hemicolectomy	18 (13.0)	20 (31.7)	15 (29.4)	
Left hemicolectom	15 (12.6)	5 (7.9)	3 (5.9)	
Sigmoidectomy	23 (16.7)	20 (31.7)	14 (27.5)	
Rectal resection	74 (53.6)	18 (28.6)	19 (37.3)	
Hartmann	8 (5.8)	0 (0.0)	0 (0.0)	

BMI, body mass index; ASA, American society of anesthesiologists; CEA, carcinoembryonic antigen.

### Intraoperative and Perioperative Outcomes

Intraoperative and postoperative outcomes are shown in **Table 3**. The mean operation time in groups B and C, who underwent SILS surgery, was shorter than that in group A, who underwent traditional laparoscopic surgery (group A 134.0 ± 42.5 min; group B 117 ± 38.9 min; group C 111.7 ± 35.4 min, p=0.004). The estimated surgical blood loss (ml) of those who underwent SILS was less in groups B and C than in group A (group A 165.1 ± 142.2; group B 122.0 ± 79.4; group C 105.2 ± 55.8, p=0.011). The length of surgery incision (cm) was also shorter in groups B and C than in group A (group A 7.34 ± 1.05; group B 5.60 ± 0.80; group C 5.28 ± 0.52, p<0.001). In contrast, blood transfusion rate (group A 15.2%; group B 14.3%; group C 9.8%, p=0.630) and intraoperative complications like vascular injury or conversion to open surgery (p=0.623) showed no difference among the three groups.

**Table 3:** Operative data.

Parameter	CLS	SILS	SILS (ERAS)	*P* value
Total surgical time, minutes				<0.001
Mean ± SD	134.01±42.50^a,b^	115.86±37.27^b^	112.49±26.68^a^	
Estimated surgical blood loss, mL				0.004
Mean ± SD	165.07±142.17^c,d^	121.27±79.22^d^	109.41±79.41^c^	
Length of surgery incision (cm)				<0.001
Mean ± SD	7.34±1.05^e,f^	5.60±0.80^f^	5.28±0.52^c^	
Blood transfusion (cases), n (%)	21 (15.2)	9 (14.3)	5 (9.8)	0.630
Intraoperative complications, n (%)				
Vascular injury	14 (10.1)	6 (9.5)	7 (13.7)	0.623
Conversion to open surgery	0 (0.0)	0 (0.0)	0 (0.0)	:

a CLS vs SILS (ERAS),P=0.002;.

b CLS vs SILS,P=0.006.

c CLS vs SILS (ERAS),P=0.013;.

d CLS vs SILS,P=0.046.

e CLS vs SILS (ERAS),P<0.00.

f CLS vs SILS,P<0.001.

### Pathologic Outcomes

The tumor size, proximal and distal resection margins, number of harvested lymph nodes, cell type, tumor differentiation, neurovascular invasion, perineural invasion, and pathologic stage were similar among the three groups (**Table 4**). No positive circumferential resection margins were found in the cases of rectal cancer.

**Table 4:** Data related to tumor pathology.

Parameter	CLS	SILS	SILS (ERAS)	*P* value
Histology type, n (%)				0.116
Adenocarcinoma	122 (88.4)	52 (82.5)	39 (76.5)	
Others	16 (11.6)	11 (17.5)	12 (23.5)	
Tumor differentiation, n (%)				0.427
Well differentiated	13 (9.4)	10 (15.9)	9 (17.6)	
Moderately differentiated	117 (84.8)	51 (81.0)	39 (76.5)	
Poorly differentiated	8 (5.8)	2 (3.2)	3 (5.9)	
Tumor depth (T classification), n (%)				0.141
T1	13 (9.4)	7 (11.1)	6 (11.8)	
T2	32 (23.2)	13 (20.6)	17 (33.3)	
T3	46 (33.3)	29 (46.0)	20 (39.2)	
T4	47 (34.1)	14 (22.2)	8 (15.7)	
Lymph node metastasis, n (%)				0.382
No	93 (67.4)	41 (65.1)	39 (76.5)	
Yes	45 (32.6)	22 (34.9)	12 (23.5)	
TNM stage, n (%)				0.453
I	40 (29.0)	18 (28.6)	21 (41.2)	
II	57 (41.3)	23 (36.5)	18 (35.3)	
III	41 (29.7)	22 (34.9)	12 (23.5)	
Largest tumor diameter (cm)				0.118
Mean ± SD	3.96±1.81	3.65±1.39	3.45±1.07	
Lymph nodes in resected specimen, n				0.545
Mean ± SD	13.70±2.35	13.48±2.77	14.00±2.65	
Proximal margin (cm),Mean ± SD				
Colon	7.45±4.96	7.68±3.82	8.29±5.81	0.722
Rectum	7.79±3.48	5.91±1.73	7.22±3.71	0.107
Distal margin (cm), Mean ± SD				
Colon	6.41±3.69	7.16±5.76	7.54±5.16	0.519
Rectum	2.31±0.97	2.93±1.28	2.63±1.55	0.097
Lymphovascular invasion, n (%)				0.323
No	95 (68.8)	48 (76.2)	40 (78.4)	
Yes	43 (31.2)	15 (23.8)	11 (21.6)	
Perineural invasion, n (%)				0.635
No	93 (67.4)	43 (68.3)	38 (74.5)	
Yes	45 (32.6)	20 (31.7)	13 (25.5)	

TNM, tumor-node-metastasis.

### Postoperative Function Analysis

Postoperative function analysis was performed according to the surgical procedures as shown in **Table 5**. The time before first flatus (hours) in group C, who underwent SILS and ERAS was shorter than in groups A and B, who underwent routine preoperative preparation (group A 61.85 ± 21.14; group B 58.30 ± 20.08; group C 42.06 ± 23.72, p<0.001). Furthermore, the days prior to the administration of free oral fluids in group C was shorter than in groups A and B (group A 4.79 ± 1.28; group B 4.67 ± 1.11; group C 2.62 ± 0.64, p<0.001). The days prior to the resumption of solid diet was less in groups A and B (group A 7.22 ± 3.87; group B 7.08 ± 3.18; group C 5.75 ± 1.70, p=0.027). The postoperative LOS (days) was also less in groups A and B (group A 9.46 ± 4.84; group B 9.52 ± 7.45; group C 7.20 ± 2.37, p=0.023); The VAS scores in days 0, 1, and 2 in groups B and C, who underwent SILS were lower than group A, who underwent traditional laparoscopic surgery (day 0 p<0.001; day 1 p<0.001; day 2 p=0.002), while the VAS score in day 3 showed no differences among the three groups (group A 1.29 ± 1.38; group B 0.98 ± 1.24; group C 0.75 ± 0.64, p=0.018). The 30-day mortality postoperative rate was zero in the three groups.

**Table 5:** Data related to postoperative function.

Parameter	CLS	SILS	SILS (ERAS)	*P* value
Duration before first flatus (hours)	61.85±21.14^a^	58.30±20.08^b^	42.06±23.72^a,b^	<0.001
Days prior free oral fluids (days)	4.79±1.28^c^	4.67±1.11^d^	2.62±0.64^c,d^	<0.001
Duration prior solid diet (days)	7.22±3.87^e^	7.08±3.18	5.75±1.70^e^	0.027
Postoperative length of stay (days)	9.46±4.84^f^	9.52±7.45^g^	7.20±2.37^f,g^	0.023
VAS score				
Day 0	3.14±2.08^h^	2.43±1.48^i^	1.71±0.88^h,i^	<0.001
Day 1	3.72±1.62^j^	3.22±1.49^k^	2.24±0.97^j,k^	<0.001
Day 2	2.43±1.52^m^	1.98±1.37	1.67±0.93^m^	0.002
Day 3	1.29±1.38^n^	0.98±1.24	0.75±0.64^n^	0.018
30-day mortality, n (%)	0 (0.0)	0 (0.0)	0 (0.0)	

VAS,Visual analogue scale.

a,c,e,f,h,j,m,n CLS vs SILS (ERAS) b,d,g,I,k SILS vs SILS (ERAS).

a P<0.001;

b P<0.001;

c P<0.001;

d P<0.001;

e P=0.025;

f P=0.009;

g P=0.020;

h P<0.001;

i P=0.030;

j P<0.001;

k P<0.001;

m P=0.001;

n P=0.007.

## Discussion

The ERAS program has been widely combined with laparoscopic colorectal surgery using a multimodal stress-minimizing approach to reduce perioperative stress, maintain postoperative physiological function, accelerate recovery after surgery, reduce rates of morbidity, improve recovery, and shorten the LOS after a major colorectal surgery (15–18). SILS for CRC was first reported in 2008 and developed rapidly in recent years in both the number and type of operations and the type of operation; however, compared with CLS, the safety and radical effects showed no difference in SILS CRC, while the latter showed potential benefits of reducing postoperative pain and better cosmetic effects, which were performed by experienced surgeons (19–25). The combination of ERAS and SILS in CRC may have a synergistic effect on the recovery of patients. Min Ki Kim et al. reported that an ERAS program combined with SILS showed early dietary resumption, shorter hospital stays, and appropriate control of postoperative pain without increases in complications or readmission rates in CRC patients compared to a conventional perioperative care with laparoscopic CRC surgery (14). However, patients with rectal, descending colon, and transverse CRCs were excluded.

In this study, we found that the median operation time was shorter in the SILS and SILS + ERAS group than in the CLS group. There may be two reasons for this result. First, the patients who underwent SILS had another set of exclusion criteria: (1) cT4b; and (2) tumor diameter ≤ 4 cm. Second, all SILS were performed with a 3D laparoscope and flexible laparoscope, which was not applied in the CLS group.

The total incision length is often used in the evaluation of cosmetic effects. In this study, the SILS ± ERAS group had a shorter incision length because of fewer trocars. However, the cosmetic effect is a subjective feeling that is not only determined by the incision length. Some reported scales and questionnaires may be more suitable for evaluating cosmetic effects. The SILS ± ERAS group showed lower VAS scores on postoperative day 0, 1, and 2 with similar postoperative analgesic usage, which may be related to fewer incisions. The VAS score and cosmetic effect evaluation will affect the postoperative psychological recovery. The pathologic outcomes showed no differences among the three groups, and the radical effect was reliable in the SILS ± ERAS group.

The recovery process in the SILS+ERAS group was significantly faster compared to the SILS and CLS group, including the time before first flatus, days prior to administration of free oral fluids, days prior to resumption of solid diet, and the postoperative LOS (26).

At present, SILS and ERAS programs have rapidly developed worldwide for CRC. Although the SILS technology is mainly limited by the technical challenges, in the future, with the integration of instrument functions and the application of robotic surgery, the difficulty of SILS will be further reduced, and it will be popularized and applied more widely. Furthermore, the combination with ERAS may be a priority for the appropriate patients, which can reduce the hospitalization time and cost of hospitalization, and obtain better cosmetic effects and psychological rehabilitation.

This study had several limitations. First, this was a small retrospective study. Therefore, selection bias could not be excluded. However, this bias was minimized by selecting study subjects with the same eligibility criteria from the two different data sets. Second, all SILS were performed by the same senior surgeon, but the CLS was performed by six different surgeons, which may have led to a bias in operation time, therapy after surgery, and LOS. However, the bias was small and the same in different groups. Third, another group of CLS+ERAS cases may need to be more convincing. In fact, the number of these cases was very small, so they were not included in the statistics, but it did not affect the conclusion of the study.

## Conclusions

These findings suggest that SILS combined with ERAS may be a feasible and safe procedure for CRC surgery because it provides favorable cosmetic results, early dietary resumption, shorter hospital stays, and appropriate control of postoperative pain without increases in complications or readmission rates. Further prospective randomized controlled studies are needed to enhance evidence-based medical evidence.

## Data Availability Statement

The original contributions presented in the study are included in the article/Supplementary Material. Further inquiries can be directed to the corresponding authors.

## Author Contributions

CW and HF conceived of the study, and prepared the manuscript draft. TZ, KL, and RZ critically revised the manuscript for important intellectual content. XZ, ZS, YL, YS, YJ, and XC performed the data collection. All authors contributed to the article and approved the submitted version.

## Funding

This study was supported by Advanced and Appropriate Technology Promotion Projects of Shanghai Municipal Health Commission (2019SY058), Clinical Skills and Innovations 3-year program of Shanghai Hospital Development Center (16CR2064B) and the Special Clinical Research Projects of Shanghai Municipal Health Commission (20204Y0006).

## Conflict of Interest

The authors declare that the research was conducted in the absence of any commercial or financial relationships that could be construed as a potential conflict of interest.

## Publisher’s Note

All claims expressed in this article are solely those of the authors and do not necessarily represent those of their affiliated organizations, or those of the publisher, the editors and the reviewers. Any product that may be evaluated in this article, or claim that may be made by its manufacturer, is not guaranteed or endorsed by the publisher.
